# Short-Term and Long-Term Outcomes of a Vocational Rehabilitation Program for Patients with Acquired Brain Injury in The Netherlands

**DOI:** 10.1007/s10926-017-9738-6

**Published:** 2017-11-14

**Authors:** Caroline H. van Dongen, Paulien H. Goossens, Inge E. van Zee, Kirsten N. Verpoort, Thea P. M. Vliet Vlieland, Judith M. van Velzen

**Affiliations:** 1 Rijnlands Rehabilitation Centre, PO Box 176, 2300 AD Leiden, The Netherlands; 2 Department of Orthopaedics, Physiotherapy and Rehabilitation, Leiden University Medical Center, Leiden, The Netherlands; 3 Department of Research and Development, Heliomare, Wijk aan Zee, The Netherlands

**Keywords:** Brain injury, Stroke, Traumatic brain injury, Vocational rehabilitation, Return to work

## Abstract

*Purpose* To describe short-term and long-term work status after a vocational rehabilitation (VR) program in patients with acquired brain injury (ABI) in the Netherlands. *Methods* Patients with ABI who participated in a VR program between 2007 and 2010 were included in this study. The 4-month VR program included a multidisciplinary assessment, three meetings with all stakeholders and reintegration with coaching on the job. Short-term results at the end of the VR program were based on data extracted from medical records. Long-term results were determined at 3–6 years (mean 4.4 years) after the program based on patient-reported data. Outcome measures included return to work, hours at work and task adjustments. *Results* Fifty-eight patients were included [mean age 48 (SD 9.4) years; n = 33 male; all working before ABI]. After the intervention, 50 patients (86%) had returned to work, working on average 60% of their former hours. Working tasks were adjusted in 48 patients. At long-term follow-up 28 patients had paid work, working on average 5.3 h more than immediately after the VR program. *Conclusions* Directly after the intervention 86% of the patients had returned to work. After 3–6 years, 64% of these patients were still working in a paid job.

## Introduction

People with acquired brain injury (ABI), including both traumatic brain injury (TBI) and non-traumatic brain injury (NTBI) such as stroke, often have difficulties returning to work [1]. For these people, return to work (RTW) is important, since it improves self-confidence and quality of life. Additionally, RTW has extended economic and societal value and it is one of the important goals in rehabilitation [2–5]. In reviews published in the past 10 years, RTW rates after ABI varied widely, from 12 to 87% [6, 7]. A mean RTW rate of 40% within 2 years after ABI was reported [1]. A recent study in Sweden reported a RTW rate of 75% after a follow-up of 6 years in patients registered with a first ever clinical stroke [8].

Vocational rehabilitation (VR) is commonly provided to reduce work disability after ABI. Several methods or models to provide VR have been described [9–12]. These methods include work-directed intervention components, education and coaching, skills training, cognitive rehabilitation, supported employment or combinations [12].

Multiple studies showed that VR has a positive effect on RTW rate after ABI [9–17]. For example, a study that evaluated the effect of a workplace intervention program after stroke reported a RTW rate of 60% at 6 months after stroke [13]. In two studies investigating the effect of resource facilitation services after ABI, with a priority on assisting patients in returning to work, 64 and 69% of the patients returned to work or school [14, 15]. Furthermore, a study reported that 68% of the participating TBI patients returned to work after 22 months of supported employment [16]. In a study of a VR program in the UK, that combines many elements of different methods of VR, 41% of the participants were discharged into paid competitive employment [17]. However, there are concerns about long-term stability of employment after ABI. For example, one study examining the history of work retention of TBI patients showed only 46% maintained stable, uninterrupted employment 3–5 years post injury [18].

In a Dutch rehabilitation centre a 4-month outpatient VR program with work-directed interventions, education and coaching on the job has been used for several years as usual care for ABI patients with a care need on this subject. Until now, the results of this so-called “round table VR program” are unknown. This study aims to evaluate the results of this VR program for people with ABI on RTW. Given the importance of maintaining employment and most studies only describing short-term results, this study also focusses on the long-term outcomes. The main research questions are ‘how many patients have returned to work after the VR program?’ and ‘how many patients are employed 3–6 years after the VR program?’. Since task adjustments are common [1], and change in number of hours at work has been described as an important outcome measure [19], these aspects are evaluated as well.

## Methods

### Study Design

In this descriptive cohort study, the effect of a VR program was evaluated by chart review and a follow-up questionnaire. The so-called “round table VR program” was already part of usual care in the Rijnlands Rehabilitation Centre, Leiden, the Netherlands. The study falls outside the remit of the Medical Research Involving Human Subjects Act (Medical Ethical Review Board of the LUMC, Leiden, the Netherlands) and was performed in accordance with the ethical standards laid down in the 2013 World Medical Association Declaration of Helsinki amendment [20].

### Participants

All patients with ABI who participated in the round table VR program of the Rijnlands Rehabilitation Centre, Leiden, the Netherlands, between January 2007 and May 2010 were identified from the patient registry of the rehabilitation centre. Patients were referred to this VR program when expressing a care need for VR after ABI. Inclusion criteria for the VR program were: having non-progressive ABI, being employed before ABI, motivated to get back to work and having an employer willing to participate in the VR program. Exclusion criteria were: being dependent on other people in activities of daily living, having a mental status that prohibits normal social contacts with colleagues or having a physical status interfering with the possibility to travel to or perform work. Additionally, patients were excluded for this study if they had attended only one of the three planned meetings (see description of the program in ‘intervention’).

### Procedure

After identifying the participants from the patient registry, data of the short-term outcomes (defined as the outcomes directly after the end of the VR program) were collected by investigating medical records, the reports of neuropsychological assessments and the minutes of the three meetings related to the intervention. A data extraction form was used. All data were retrieved by one person, not involved in the program (IZ). Data extraction from a random sample of five sets of records, analysed by a second assessor (PG), revealed no relevant differences.

For long-term follow-up, in June 2013 (3–6 years after the end of the patients’ VR programs) all patients were sent a questionnaire by mail that had been self-designed by the investigators to gather information about work status and hours at work (quantitative data). Questions about task adjustments and, if applicable, reasons for not continuing to work in a paid job were included to collect qualitative data. If the questionnaire was not returned within 3 months, patients were contacted by phone as a reminder. To increase the response rate to accurately answer our main research question, patients who had not returned the questionnaire after the reminder were contacted again by phone. Depending on the patients preference and with explicit permission of the patient, either the full questionnaire was completed by phone or the patient just answered the main research question, i.e. performing paid work or not.

### Intervention

Patients took part in a 4-month multidisciplinary VR program. The professionals involved in the multidisciplinary team included the rehabilitation specialist, occupational therapist, social worker, neuropsychologist and, if needed, a speech therapist (in case of aphasia or dysarthria) or physical therapist (in case of work-interfering motor problems). The team collaborated actively with the other stakeholders of the patient’s RTW process, including the patient and his or her partner/important other, the employer, the occupational physician and a co-worker. The co-worker was usually a colleague close to the patient and his work, able to give feedback to the patient on work performance. Before the start of the program all patients gave written permission for an open exchange of information between the stakeholders. The minutes of all meetings were sent to all stakeholders.

The program consisted of six steps involving all stakeholders including three “round table” meetings:

1. The rehabilitation specialist considered eligibility of the patients for the VR program based on previous mentioned in- and exclusion criteria. During an initial assessment, involving the neuropsychologist and occupational therapist, the patient’s (dis)abilities and wishes related to RTW were identified. Subsequently, the job characteristics and demands were determined. Abilities and disabilities of the patient were determined in accordance with the International Classification of Functioning, Disability and Health (ICF model) [21].
2. During the first round table meeting, which marks the official start of the VR program, all stakeholders were informed on the disease and the abilities and disabilities of the patient by the rehabilitation team members. Thereafter, the demands, expectations and possibilities for RTW were discussed. Consensus and commitment between all stakeholders on the first steps of reintegration with regard to working hours and tasks were obtained.
3. Task-oriented rehabilitation training was provided on the job with the support of a co-worker. The occupational therapist weekly gave individualized support to the patient, either in the rehabilitation centre or on the job. The occupational therapist contacted the co-worker every 2 weeks by telephone or more often if necessary. During these contacts the progress was evaluated and advice was given about an increase in working hours and tasks. The neuropsychologist was in contact with the patient every 2–4 weeks. The social worker contacted the patient and his or her partner/important other every 2 weeks.
4. 8 weeks after the start of the VR program the second round table meeting took place. In this meeting all stakeholders described their experiences: the problems that were experienced during the training weeks were discussed, as were the things that went well. The increase in working hours, the expansion of tasks and the bottlenecks were discussed. At the end of this meeting a decision was made on the continuation of the VR program and adaptations regarding working hours and tasks.
5. When VR proceeded, the patient continued the task oriented rehabilitation training on the job by a further increase of working hours and tasks, supported by the co-worker and the occupational therapist. Contact with the social worker and neuropsychologist continued.
6. 16 weeks after the start of the VR program the final round table meeting took place. The main subject of this meeting was to discuss the prognosis for RTW with respect to achievable working hours and tasks. The VR program provided by the professionals of the rehabilitation centre stops after the meeting. Further vocational reintegration and on-going support were referred to the occupational physician, which is usual care in the Netherlands.

### Variables

#### Baseline Characteristics

Socio-demographic characteristics assessed at the start of the VR program were age, gender, social status (‘married/cohabiting’ or not) and education level (‘university/higher education’, ‘professional training/high school’ or ‘occupational training or less/primary school’). The underlying diagnosis of ABI, the time since injury and total duration of rehabilitation were documented. It was registered whether patients had received inpatient rehabilitation (yes/no) prior to their outpatient rehabilitation. Physical and language functions were assessed using four items selected from the National Institutes of Health Stroke Scale (NIHSS) [22]. These items were: limb ataxia, sensory deficits, dysarthria and aphasia and were categorized as present or not. Arm activities were scored as limited (varying from only limitations in fine motor skills to no activities possible) or not (bimanual activities without limits). Further disease characteristics assessing functioning included the ‘Functional Ambulation Categories’ score [FAC; 0 (non-functional ambulator)—5 (independent ambulator who can walk freely on any surface) points [23]], ‘Test of Sustained Selective Attention’ (TOSSA; expressed as a score between 0 and 100%, with a score lower than 74.4% interpreted as having a disturbance in attention [24]) and ‘Tower of London Test’ (TLT, expressed as a score between 0 and 100%, with a score lower than 56.9% interpreted as having a disturbance in planning or executive functioning [25, 26]). The number of hours that patients worked prior to the ABI and at the start of the VR program were documented. The size of the company was recorded and categorized in companies with ≤ 100 or > 100 employees. The physical and mental job demands were recorded as blue collar (involving mainly physical labour) or white collar (including mainly professional, managerial or administrative work).

#### Outcome Measures

Outcome measures were RTW, hours at work and task adjustment. For the short-term analysis RTW was defined as ‘performing useful tasks for the employer’. For the long-term analysis RTW was defined as ‘performing paid work yes/no’. Hours at work and task adjustment directly after the program were extracted from the minutes of the meetings in the medical records and therefore determined by the VR team and the employer. At follow-up these outcomes measures were self-reported by the patient. Hours at work did not include hours spent on volunteer work.

### Analysis

All statistic analyses were performed using SPSS 19 software package (SPSS Inc., Chicago, IL). Descriptive statistics (i.e., frequencies and percentages) were used for quantitative data in relation to RTW. The Wilcoxon signed-ranks-test was used to compare the number of hours at work before, immediately after the VR program and at follow-up. Significance was set at p ≤ 0.05. Qualitative data (e.g. reasons for no RTW or not maintaining employment) were grouped and summarised.

## Results

### Participants

The registry of patients who took part in the VR program from 2007 until 2010 contained 68 patients. For two patients the goal of VR was returning to school rather than to work. Of the 66 eligible patients, eight were excluded from the present study as only one of the three planned meetings took place. In four of these patient only one meeting took place because of severe behavioural disorders, prohibiting normal social contacts with colleagues. In the other four, successful RTW was attained without need for more than one meeting.

Patient characteristics at baseline of the 58 included patients are described in Table 1. Just over half of the patients were male. The mean age was 48 years. The majority of patients had had a stroke (66%) and there were eight patients (14%) with TBI. Mean time since injury in the total group was 9.0 (SD 6.8) months. Prior inpatient rehabilitation had been provided in case of 20 patients with an average duration of nearly 2 months. Three patients were impaired in ambulation activities, one-third of the patients suffered from impaired arm activities. More than one-third suffered from aphasia or dysarthria. The 58 patients were working 33.8 (SD 6.9) hours a week on average before ABI.

**Table 1:** Patients characteristics at the start of vocational rehabilitation

Total number of patients	58
Patient characteristics	
Age in years; mean	48 (SD 9.4)
Male gender	33 (57%)
Married/cohabitating	46 (79%)
Education level	
University/higher education	22 (38%)
Professional training/high school	26 (45%)
Occupational training or less/primary school	10 (17%)
Diagnosis	
Stroke	38 (66%)
Contusion or commotion cerebrum	8 (14%)
Infection	3 (5%)
Hypoxic brain injury	6 (10%)
Tumour/vascular disease	3 (5%)
Disease characteristics	
Time since injury in months; mean	9.0 (SD 6.8)
Deficits in body functions	
Limb ataxia	10 (17%)
Sensory deficit	11 (19%)
Aphasia	17 (29%)
Dysarthria	7 (12%)
Impaired walking ability, FAC < 5	3 (5%)
Impaired motor arm function	19 (33%)
Cognitive functions	
TOSSA; mean (of in total 54 patients)	78.1 (SD 20.8)
TOSSA < 74,4%	17 (31%)
TLT; mean (of in total 55 patients)	76.0 (SD 14.1)
TLT < 56,9%	4 (7%)
Job characteristics	
Employment sector: blue collar	27 (47%)
Company size: > 100	39 (67%)

All values are numbers (%) of patients, unless stated otherwise

*FAC* functional ambulation categories, *TOSSA* Test of Sustained Selective Attention, *TLT* Tower of London Test

### VR Program

For all patients, the employer, co-worker and rehabilitation team were involved in the VR program. The occupational physician was involved in the program of 40 (80%) patients with successful RTW and five (63%) of the eight patients that did not RTW. The partner or important other was involved in the program of 37 patients (74%) that did RTW and seven (88%) of the patients that did not RTW.

### Short-Term Results of VR Program

**Fig. 1:**
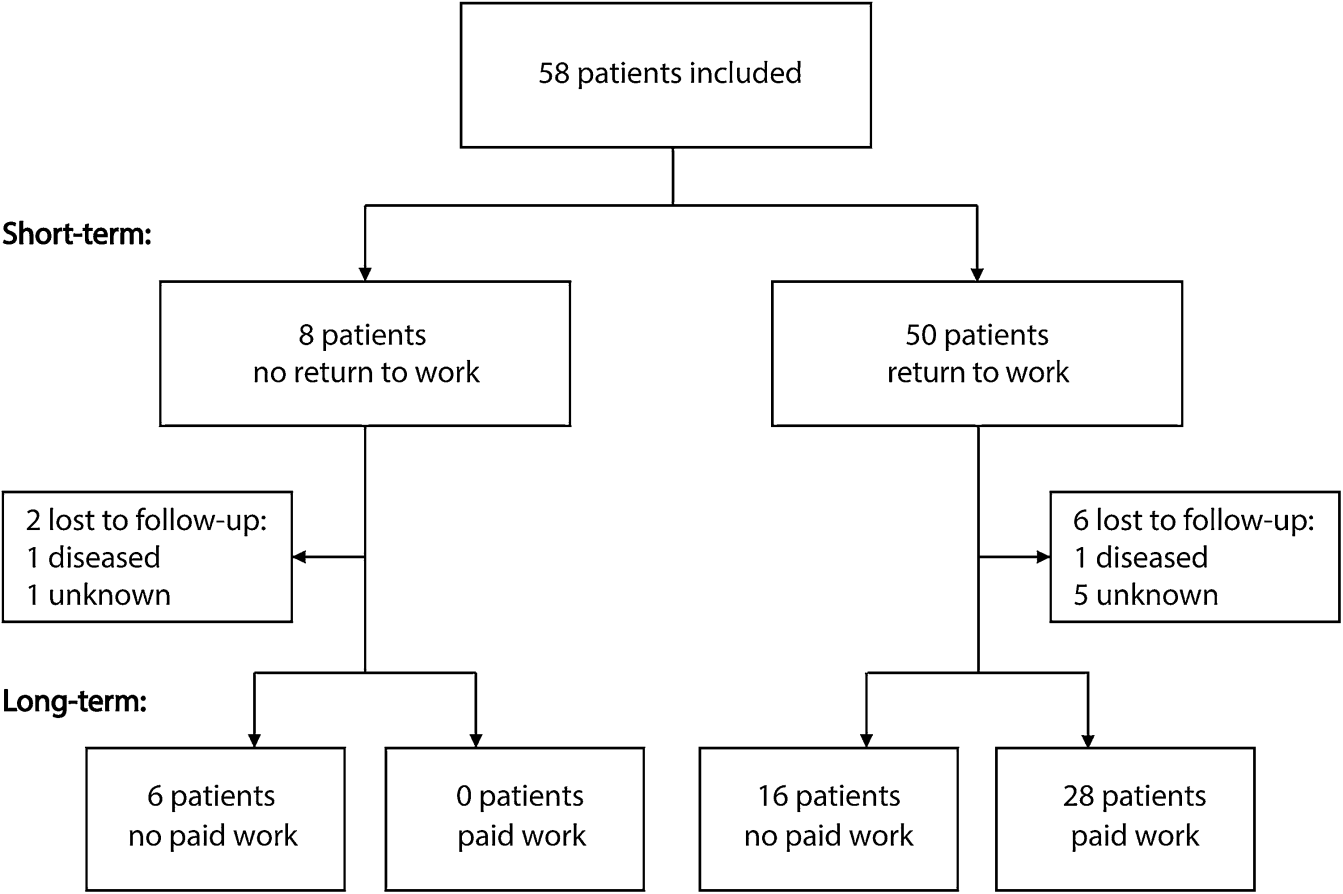
Selection and follow-up of patients

At the start of the VR program 15 (26%) patients had started working, working on average 11.1 (SD 7.5) hours per week at that point. At the end of the 4-month program 50 patients (86%) had returned to work and eight (14%) had not (Fig. 1). All of the patients that had already started working at the start of the program, were working at the end of VR. Reasons for unsuccessful work reintegration were: elimination of the job during reorganization (n = 1); cognitive functioning disorder (n = 2); low physical tolerance (n = 3) and inability to adjust tasks by employer (n = 2). In three of these eight patients the VR program was stopped after the second meeting.

Among the 50 working patients, working tasks were adjusted for 48 (96%) patients. The group of patients who returned to work at the end of the VR program (n = 50) worked on average 21.0 (SD 8.0) hours per week at the end of the VR program, which was about 60% of their former hours.

### Long-Term Results of VR Program

Follow-up data were collected 3–6 years after the VR program, with an average time to follow-up of 4.4 (SD 0.9) years. At that time, 50 patients (86% of the 58 patients initially included in this study) were able and willing to participate, two patients had died and six patients were lost to follow-up (Fig. 1).

**Fig. 2:**
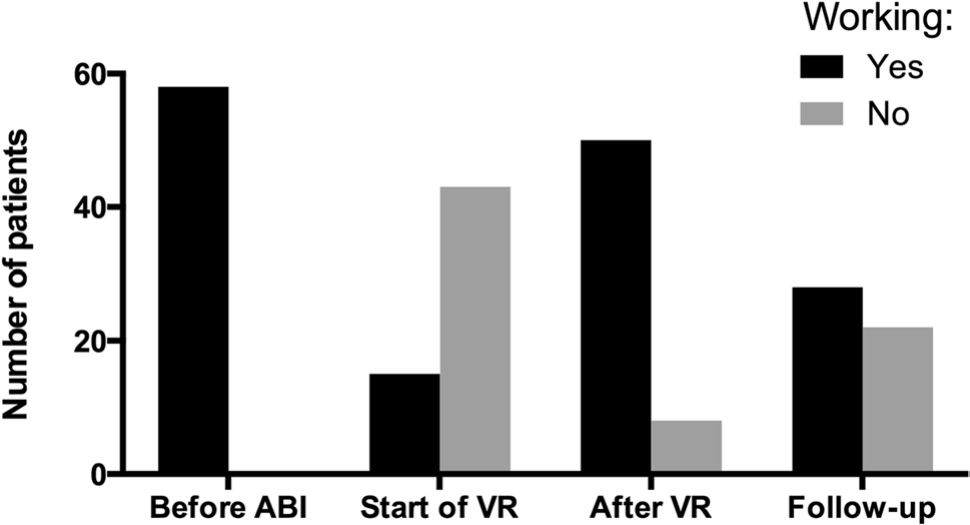
Number of patients working before ABI, at the start and end of VR and at 3–6 year follow-up. *ABI* acquired brain injury, *VR* vocational rehabilitation. At start of VR and after VR, working is defined as ‘performing useful tasks for employer’. Before ABI and at follow-up, working is defined as ‘performing paid work’

At follow-up 28 (64%) of the 44 patients who were working after the VR program reported to work in a paid job. None of the patients who did not RTW after VR reported to work at long-term follow up (Fig. 1). Patients were working on average 26.5 (SD 10.8) hours per week (Fig. 2). On average, this is 5.9 h less per week than these 28 patients worked before ABI (p < 0.01), and 5.3 h more than immediately after the VR program (p < 0.01). Of these 28 patients, four patients reported to work the same number of hours as before ABI and six patients reported to work more hours than before ABI. Patient-reported task adjustments were inadequate to be reported, since there was a substantial amount of missing data and inconsistent responses within the questionnaires were found in multiple cases.

Of the 16 patients that were working directly after VR, but were not working in a paid job at follow-up, five reported reasons that did not relate to their work disabilities after ABI. Those reasons included elimination of their job during reorganization in a time of economic recession (n = 3), early retirement (n = 1) and not being able to work due to a malignancy (n = 1).

## Discussion

This descriptive study shows the short-term and long-term outcomes on RTW, number of hours at work and task adjustments of a so-called “round table VR program” for ABI patients. At the end of the VR program 86% of the patients with recent ABI had returned to work. About two-thirds of them were able to keep working at the long term.

The number of people that had returned to work at short-term follow-up is relatively high compared to the results of three reviews on the evaluation of several VR interventions with RTW rates varying between 38 and 85% [10–12]. Since most earlier performed studies only reported follow-up outcomes up to 6 months or 1 year after VR [12], it is difficult to compare the long-term results of this study. One study of a holistic cognitive rehabilitation program after TBI with guided occupational trials reported that 50% of the patients that were followed up until 3 years after the program were employed competitively [27]. In a follow-up study in which only patients that had returned to work after rehabilitation were selected about two-thirds of the patients had been working competitively for many years, as reported 7 years after the rehabilitation program [28]. Another follow-up study reported that 32% of those working at 2 years after comprehensive rehabilitation were not employed at 5 years [29]. These numbers correspond quite well with the results of this study, in which 64% (28 of the 44 patients who were working after VR) reported to be working in a paid job on average 4.4 years after VR.

At the start of the VR program some patients (26%) had already started working, which could have contributed to the high success rate. Another possible explanation for the high RTW rate might be the involvement of all stakeholders in the round table VR program: the patient, the multidisciplinary rehabilitation team, the partner/important other, the occupational physician, the employer and a co-worker. All participants were informed about the impairments and possibilities of the patient with ABI in the same manner and at the same time. This is in concordance with the Dutch Guideline on ABI and work participation, based on both literature and expert opinion. This guideline advises active commitment of all participants, equal information and a shared goal to RTW for all stakeholders [30]. Also, the Dutch law states that both the employer and the employee are responsible for the RTW process during the first 2 years of sick leave. The employer is required to make an effort to provide (temporary) modified work to facilitate RTW. After 2 years, the contributions of the employer and employee on the RTW process are assessed by an insurance physician and it is assessed whether the employee qualifies for a government disability allowance [31]. Because short-term follow-up was within the first 2 years after sick leave for all patients except one and long-term follow-up was after those first 2 years, the Dutch legislation could be an explanation for the high initial RTW rate and of the difference in RTW rate between short-term and long-term follow-up.

This current study is the first to report detailed changes in hours at work after ABI. The majority of patients required a reduction in the number of hours at work. In this study 64.3% (18 of the 28 patients who were working at 3–6 years follow-up) worked fewer hours than before ABI at long-term follow-up. This does not completely correspond with the results of a study comparing employment 1 year after TBI between men and women, in which 9.5% of the patients worked more hours 1 year post injury, 36.5% worked the same number of hours as pre injury, 12.9% worked fewer hours and 41.4% had stopped working [19]. Another interesting finding in the current study is that patients were working on average 5 h more at follow-up than directly after the VR program. Patients who were able to maintain employed, were able to further increase their working hours.

Nearly all patients needed working task adjustments in this study. This is in concordance with the outcome of the review of Van Velzen et al. [1] in which it was described that changes of occupational and job demands are common after ABI. Another review aimed at determining effective RTW interventions pointed out that adaptation of the working tasks is even an important component of successful VR programs [12].

### Limitations of the Study

A limitation of this study is that different definitions of RTW for short-term and long-term follow-up were used. RTW at the end of the VR program was defined as ‘performing useful tasks for the former employer’, which does not reflect earning capacity. However, according to the Dutch law, during the first 2 years of sick leave the employer is responsible for the cost of wage-replacement [32], making an outcome measure looking at earning capacity less suitable at that time point. At follow-up at 3–6 years after the VR program it was chosen to define RTW as ‘performing paid work’ to specify the outcome measure to facilitate comparing the results with other VR programs and literature. Another limitation of this study which needs to be noted is that long-term follow-up was not set at one time point but varied between 3 and 6 years due to practical reasons, which makes comparisons more difficult.

This study only shows the results of VR of patients that met the specific in- and exclusion criteria of the VR program, including being employed prior to ABI and not being dependent on other people in activities of daily living. Therefore, the results of this study can not be generalised to all ABI patients. However, our study population consisted of a heterogeneous group with variable disabilities and this study reflects common practice in our rehabilitation centre, which is a strength of the study. As suggested in literature, both patients with TBI and NTBI were considered as one population [12]. Since Dutch legislation might have influenced the results of this study, mainly the short-term RTW rate, our results can not be directly extrapolated to other countries.

### Recommendations

A VR program that involves all stakeholders and provides training on the job seems to be a successful intervention. The difference in work status directly after VR and several years later emphasizes the importance of providing long-term follow-up data of VR programs. Future research could focus on interventions that are also aimed at maintaining employment.

## Conclusion

This study describes a VR program that includes a multidisciplinary assessment, three meetings with all stakeholders and reintegration with coaching on the job for patients with ABI. Eighty-six percent of the patients were able to perform useful activities for their employer directly after the VR program. Working tasks were adjusted for 48 (96%) patients. At 3–6 years after the intervention, 64% of the patients who were working after the VR program reported to perform paid work. These patients were working on average 5 h more than directly after the VR program.
